# Optimization of Brewer’s Yeast Quantity in Liquid and Gel Larval Diets for the Mediterranean Fruit Fly

**DOI:** 10.3390/insects14100828

**Published:** 2023-10-21

**Authors:** Paraschos N. Prekas, Vasilis G. Rodovitis, Evmorfia P. Bataka, David Nestel, Christos T. Nakas, Nikos T. Papadopoulos

**Affiliations:** 1 Laboratory of Entomology and Agricultural Zoology, Department of Agriculture, Crop Production and Rural Environment, School of Agricultural Sciences, University of Thessaly, Fytokou St., 38446 Volos, Greece; parprekas@uth.gr (P.N.P.); rodoviti@uth.gr (V.G.R.); 2 Laboratory of Biometry, Department of Agriculture, Crop Production and Rural Environment, University of Thessaly, Fytokou St., 38446 Volos, Greece; bataka@uth.gr (E.P.B.); cnakas@uth.gr (C.T.N.); 3 Institute of Plant Protection, Department of Entomology, ARO, The Volcani Ctr., Beit Dagan 50250, Israel; nestel@agri.gov.il; 4 University Institute of Clinical Chemistry, Inselspital, Bern University Hospital, University of Bern, 3010 Bern, Switzerland

**Keywords:** medfly, Tephritidae, fitness, rearing, liquid diet, gel diet, adult performance

## Abstract

**Simple Summary:**

Management of the Mediterranean fruit fly (medfly) with the application of the sterile insect technique depends on the production and release of high quality sterile males that can compete with the feral males to induce sterility in the wild population. Larval rearing should be cost-effective and practical. The right balance between the cost of the protein source (the most expensive ingredient) in larval diets and the quality of the adults produced is a key requirement for effective and economically viable rearing. Two types of diet, a liquid and a gel one, were evaluated across different brewer’s yeast concentrations (protein source) based on survival and developmental duration of larvae and survival of adults. Overall, regardless of the type of diet, brewer’s yeast reduction to 50% of its initial quantity did not affect the quality of the adults produced. Although the performance of immature medflies from the gel diet was inferior compared with the liquid diet, an interesting range of developmental duration phenotypes was observed, opening interesting questions for future experimental work.

**Abstract:**

Several artificial larval diets have been developed, evaluated and used for mass-rearing of the Mediterranean fruit fly (medfly), *Ceratitis capitata* (Wiedemann) (Diptera: Teprhitidae). There are several efforts to reduce the cost of rearing and optimize the quality of the produced sterile males that are destined for release in sterile insect release programs. Survival, growth, longevity and reproductive capacity of sterile males are strongly connected with the most expensive ingredient, the brewer’s yeast (protein), in the larval diet. The current study focused on settling the optimal content of brewer’s yeast in a liquid diet and a gel diet. Egg hatch rates, developmental duration of immatures, pupation rate, pupae and adult survival were recorded as indicators of quantity and quality of the produced adults. Egg hatch was higher and larval developmental duration longer in the gel diet. In contrast to the liquid diet, an increase in brewer’s yeast concentration was correlated with increased pupation rate and pupae survival in the gel diet. Reducing brewer’s yeast up to 50% of its initial quantity had no significant effect on the survival of the emerging adults regardless of the diet type. Our findings may contribute to the production of low-cost and effective diets for use in mass-rearing facilities of medflies.

## 1. Introduction

Nutrition is a fundamental determinant of insects survival, profoundly impacting their development, growth, behavior and ecology [1,2]. In fruit flies (Diptera: Tephritidae), nutrition affects development and fitness [3,4,5], and deficiency or absence of specific nutritional components such as proteins, carbohydrates and vitamins in food are negatively correlated with survival, developmental duration and fitness [6,7,8]. The nutritional environment of larvae may greatly affect body size [9,10], and the life history of tephritids, including adult survival and reproduction [10,11]. The size of adults, especially that of males, is associated with mating success [12], and hence, the selection of a nutritionally proper larval diet is considered of major importance in autocidal control programs, such as the sterile insect technique (SIT), that rely on male mating performance to succeed [11,13,14].

The Mediterranean fruit fly (medfly), *Ceratitis capitata* (Wiedemann) (Diptera: Tephritidae) is as a highly polyphagous pest that can infest more than 300 different hosts and is considered one of the most threatening pests of the fruit producing industry worldwide [15,16]. Because of its economic importance, there are intensive efforts to develop effective control methods and strategies to manage medfly populations [17,18,19]. For example, the sterile insect technique (SIT) has been extensively applied against medfly [20].

SIT is an environmentally friendly control method involving mass-rearing, sex separation and sterilization of males [21]. Sterilized males are released in the wild and are expected to outcompete wild males, mate with feral females and induce sterility in wild populations [21]. SIT depends on mass-rearing to produce numerous, high-quality males, capable of outcompeting wild ones in the field [20]. For sterile males to outcompete their wild competitors, signaling should be performed at the same intensity, sexual pheromones of high quality on an equal diurnal pattern as wilds should be released and typical courting behavior should be expressed [22,23]. Production of numerous high-quality sterile males requires massive amounts of a suitable larval diet [20,24]. Artificial diets, imitating natural food, have been developed for a wide range of taxa including fruit flies [25,26,27]. Therefore, there are several larval diets developed, evaluated, and adopted for fruit fly rearing, aiming to produce high quality males and ensure efficient productivity of the mass-rearing facilities [28,29].

Over the last two decades, liquid, meridic and oligidic larval diets have been formulated and tested for medfly rearing [7,26,29,30]. Although larval diets consisting of yeast are more expensive, they are preferred among others as they provide high protein levels [31] along with vitamins, carbohydrates and lipids to larvae [6,24,32]. For the SIT against medfly to be effective, a successful and economically viable rearing that balances the cost of the protein source in larval diets and the quality of the produced adults should be established [20,33]. Liquid diets are quite promising in small-scale laboratory rearing, but also difficult to sustain mass-rearing in the context of an SIT program [34]. For a liquid diet to be effective, a suitable larval developmental substrate (bulking agent) should be considered. Bulking agents are materials such as paper, cotton pads, bran, sawdust that are commonly used on artificial larval diets [35,36,37,38]. Those bulking compounds absorb liquid and provide insects with diet’s nutrients [38,39]. Although liquid diets are preferable over solid diets, there are disadvantages like humidity loss and frass concentration upon the bulking agent, which could be a source of pathogens, increasing toxicity and leading to high larval mortality levels [34,40,41].

Gel diets are becoming a preferred alternative to liquid ones not only because they provide similar advantages to liquid ones, but also, they are characterized by physical consistency [42]. Gel diets are homogenous, more stable and easier to handle compared to liquid and solid diets [34]. Various ingredients such as agar, carrageenan, gelatin and pregelatinized corn starches have been used as gelling agents [27,34,42]. Agar-based gel diets used for medfly mass-rearing procedures in the past achieved higher pupal rearing efficacy [43]. Despite agar’s beneficial properties, its high cost may be a drawback for large-scale rearing facilities [44]. The amount of agar has been optimized in semi-liquid or gel diets for the rearing of *Bactrocera tryoni* [34]. Then, Pascacio-Villafan et al. [27] evaluated different gelling agents compared to agar, in yeast-reduced artificial diets using *Anastrepha ludens* as a model system. Amino acids, vitamins and sucrose are the key nutritional components for medfly larval development [6]. Yeast is the main source of amino acids and protein in larval diets that greatly affects larval performance [45,46]. Although yeast is essential for producing fit adults, it is considered as the most expensive ingredient [47]. As a result, several studies have focused on producing diets with limited but sufficient concentrations of yeast [31,47]. For example, Pascacio-Villafan et al. [48] evaluated diets consisting of different levels of nutrients, as well as proteins, concluding that nutritious diets could be modified to be cost-effective without affecting the performance of *A. ludens* adults. Developing more efficient and cost-effective diets for mass-rearing is extremely challenging and requires continuous developments and further examination.

Our study evaluated different brewer’s yeast concentrations on a standard liquid diet and a gel diet, aiming on settling the optimum concentration among those tested. The diets were evaluated based on survival and developmental duration of medfly’s immatures and survival of the produced adults. Our results may contribute to the production of low-cost and effective diets for use in mass-rearing facilities of medfly.

## 2. Materials and Methods

### 2.1. Flies and Laboratory Conditions

The experiments were carried out in the Laboratory of Entomology and Agricultural Zoology at the University of Thessaly, Greece. We used eggs from a laboratory adapted strain, named “Benakeio”, which has been maintained under laboratory conditions for more than 30 years [49].

Flies were kept in wooden-framed, wire-screened cages (30 × 30 × 30 cm) under constant laboratory conditions, 25 ± 1 °C, 65 ± 5% RH and a photoperiod of L14:D10 with photophase starting at 7:00 and ending at 21:00. Flies were provided with ad libitum access to water and standard adult diet (mixture of yeast hydrolysate, sugar and water at a 1:4:5 ratio) [50]. Females deposited eggs on the inner surface of an artificial oviposition substrate (dome) comprising a red, plastic, hollow hemisphere (5 cm Ø), which was punctured with 40–50 evenly distributed holes (1 mm Ø). Each dome was fitted into a Petri dish (5 cm Ø). A plastic cup with 0.5 mL orange juice was placed in the Petri dish. Water was added in the base of the Petri dish to maintain high humidity levels within the dome [51,52,53].

Domes were placed in rearing cages for 24 h to collect eggs for experimental needs. Collected eggs were placed on Petri dishes containing either liquid diet or gel diet (Table 1). Petri dishes of both regimes were placed into plastic containers on a layer of sterilized sand, where larvae pupated.

### 2.2. Diet Formulations

The standard liquid diet consists of 250 mL water, 50 g brewer’s yeast, 50 g sugar, 25 g soy flour, 1 g of salt mixture (calcium carbonate, copper sulfate, ferric phosphate, manganese sulfate, magnesium sulfate, potassium aluminum sulfate, potassium chloride, potassium phosphate monobasic, potassium iodide, sodium chloride, sodium fluoride, tricalcium phosphate), 4 g citric acid, 4 g ascorbic acid and 0.75 g sodium propionate [50] (Table 1A). A cotton pad (5.5 cm Ø) served as a bulking agent on the liquid diet. On the other hand, the gel diet consists of the same ingredients plus 0.2 g nipagin and 2 g agar used as gelling agent (Table 1B). We evaluated 7 different concentrations of brewer’s yeast (1, 2, 5, 10, 25, 50, and 100% of the initial standard quantity of brewer’s yeast) (Table 1) on both liquid and gel diet, while all other components were kept constant.

To prepare the liquid formulation, all ingredients (Table 1A) were mixed with 250 mL water in a blender. The homogeneous mixture for each brewer’s yeast concentration was placed in a glass vial (labbox 500 mL) with a screwed cap and refrigerated at 4 °C for 24 h before use. After 24 h, the mixture was placed on cotton pads, in a 9 cm Ø Petri dish. To prepare the gel diet, all ingredients, apart from agar, were mixed in a blender with 125 mL water. Agar was mixed with 125 mL water and was heated until reaching the boiling point. The two mixtures were blended again. The diet was poured into Petri dishes (9 cm Ø) at (25 ± 1 °C, 65 ± 5% RH) until gel formation.

### 2.3. Experimental Procedure

Using a stereomicroscope, 100 eggs were randomly selected and placed on the respective larval diet regime. Five replicates were run for each treatment (brewer’s yeast content) and each diet (100 eggs/replicate: 500 eggs/treatment). In total we used 7000 eggs for the evaluation of both liquid and gel diets. Egg hatch was recorded under a stereomicroscope 3-day post oviposition.

Egg -to-pupa developmental duration was calculated (in days) as the time from the day eggs were collected and deposited on larval diet until the day pupation observed. Larval survival was estimated by recording pupation daily. Pupae sieved out from the sand were transferred to plastic Petri dishes and placed in transparent Plexi-glass cages (20 × 20 × 20 cm) that contained adult food and water ad libitum. Pupal survival was estimated by the number of emerging adults. Dead adults were counted and removed each day until the fifth day post emergence (five-day survival).

### 2.4. Data Analysis

Binary logistic regression and the chi-squared test were used to compare egg hatching, pupation rate, pupa and adult survival rates between the two types of diet and across brewer’s yeast concentrations. Effect sizes are presented as odds ratios (ORs) with 95% confidence intervals (CI), which are the odds of an event (eggs hatching, pupation rate, pupal and adult survival) in a group of interest to the odds of the same event occurring in the group used as a reference. ORs greater than 1 indicate greater odds for the group of interest, while ORs less than 1 indicate greater odds for the reference group. Furthermore, Cox proportional hazards regression models were used to infer whether the proportion of brewer’s yeast, the type of diet and their interaction were significant predictors of immature developmental duration. Kaplan–Meier estimates were used to depict pupation progress on different brewer’s yeast concentrations between the two types of diet. The analysis was carried out using R v4.2.2 [54]. The packages used to perform the analysis and produce the graphs were stats [54] and ggplot2 [55].

## 3. Results

### 3.1. Egg Hatch

**Figure 1:**
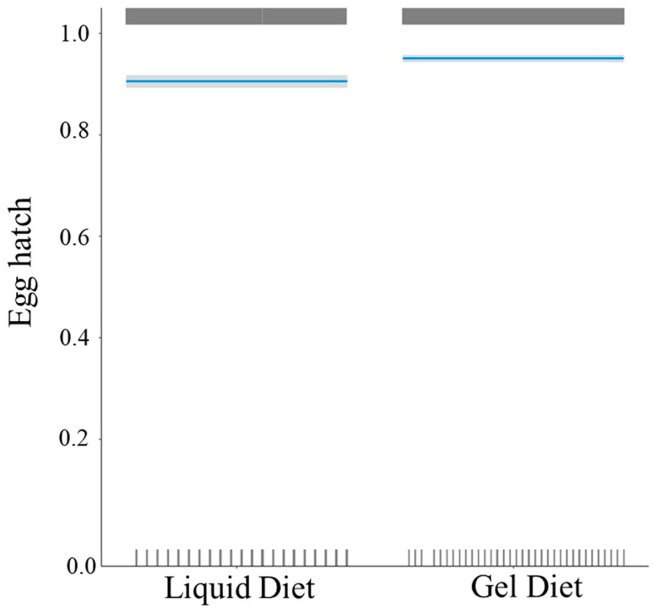
Predicted probability with 95% confidence intervals (CI) of egg hatch for both liquid and gel diet regardless of brewer’s yeast concentration. The blue lines present the predicted probability with 95% confidence intervals (CI) of egg hatch for both liquid and gel diet regardless of brewer’s yeast concentration. The upper vertical grey lines present the eggs that hatched and the lower vertical grey lines present the eggs that did not hatch in the sample for both liquid and gel diet.

Predicted probability of hatching as modeled through binary logistic regression was higher than 90% in both diets (liquid and gel). The brewers’ yeast concentration was a non-significant factor on egg hatch; thus, it was removed from the final model. Egg hatching odds increased by a factor of 2.01 for gel diet compared to liquid diet (OR (95% CI) = 2.01 (1.63, 2.49), *p* < 0.001). Gel diet had a significantly higher predicted probability (0.95) of egg hatching than liquid diet (0.90) (Figure 1).

### 3.2. Larval Developmental Time

Cox proportional hazards regression models showed that both the type of the bulking agent (Wald *χ^2^* = 1440.188, df = 1, *p* < 0.001) and the brewer’s yeast concentration (Wald *χ^2^* = 421.414, df = 1, *p* < 0.001) were significant predictors of larval developmental duration. The interaction of the aforementioned factors had a significant effect on larval developmental time (Wald *χ^2^* = 386.196, df = 1, *p* < 0.001). Overall, larval developmental duration was longer on the gel diet than on the liquid diet (*χ^2^* = 2276.8, df = 1, *p* < 0.001). All larvae completed development and pupation within 10 days from the egg collection day in the liquid diet regardless of brewer’s yeast concentration (Figure S1). However, in the gel diet, larval developmental duration exceeded 30 days on lower brewer’s yeast concentrations.

**Figure 2:**
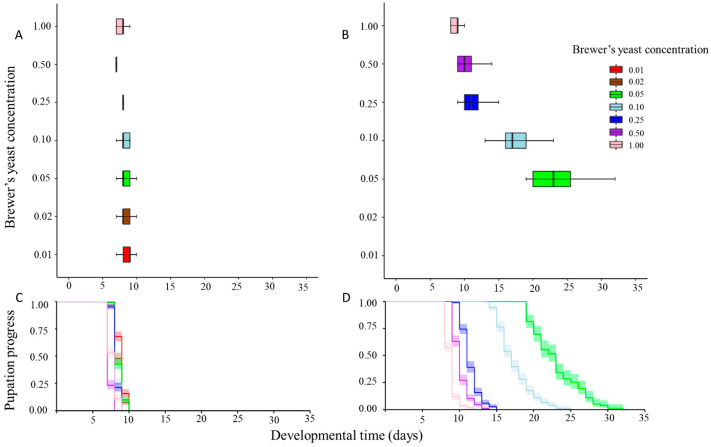
Boxplots depicting larval developmental time on liquid diet of different brewer’s yeast concentrations (**A**) and age-specific cumulative curves depicting progress of pupation with 95% confidence intervals (CI) (**C**). Boxplots depicting larval developmental time on gel diet of different brewer’s yeast concentrations (**B**) and cumulative age-specific pupation curves with 95% confidence intervals (CI) (**D**).

The progress of larval development within the different brewer’s yeast concentration for both diets is given in Figure 2. Both in the liquid diet and the gel diet, on higher brewer’s yeast concentrations, larval developmental time was shorter (*χ^2^*_liquid_ = 1492, df = 6, *p* < 0.001) (*χ^2^*_gel_ = 2047, df = 4, *p* < 0.001) (Figure 2). In the gel diet, low brewer’s yeast concentration (5%, 10%) resulted either in extended larval developmental time or pupation failure (1%, 2%), whereas in the liquid diet, larvae at lower concentrations managed to pupate.

### 3.3. Pupation Rate

**Figure 3:**
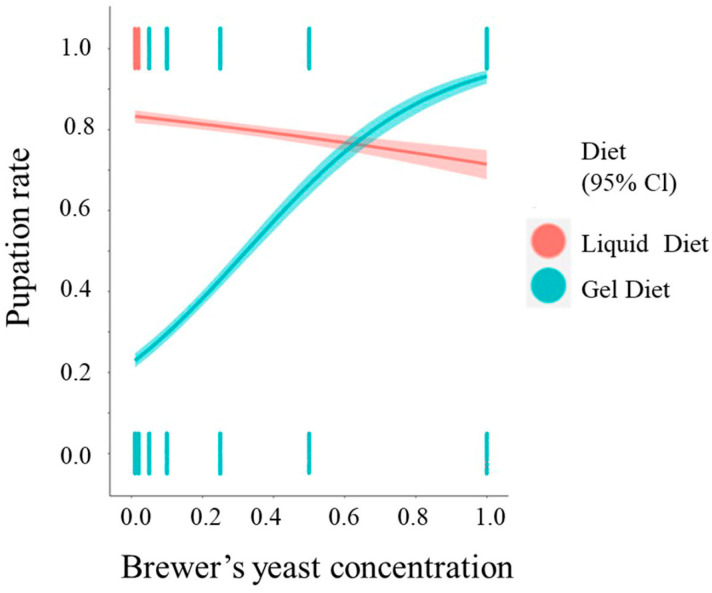
Logistic regression curves for predicted probability of pupation rate in the two types of diets with 95% confidence intervals (CI) in relation to the concentration of brewer’s yeast.

Overall, both brewers’ yeast concentration (OR (95% CI) = 0.499 (0.3966, 0.6296), *p* < 0.001) and diet type (OR (95% CI) = 0.0571 (0.0491, 0.0664), *p* < 0.001) were significant predictors of pupation (Figure 3). In the gel diet, for concentrations of brewers’ yeast lower than 5%, larvae failed to pupate. In contrast, larvae managed to pupate in each of the tested concentrations on the liquid diets. The interaction between the two predictors was significant (OR (95% CI) = 95.7 (65.1959, 141.375), *p* < 0.001). For the gel diets, the predicted probability of pupating increased with an increase in brewers’ yeast concentration. However, for the liquid diets the predicted probability decreased as the concentration of brewer’s yeast increased. The odds to pupate for each additional unit percent of brewer’s yeast for gel diets increase by a factor of 4.67% ($e^{log\left(95.7\right)\;0.01}=1.0467)$ compared to the liquid diet. The parameter estimates (ORs) of the model are displayed in Table 2.

### 3.4. Pupal Survival

**Figure 4:**
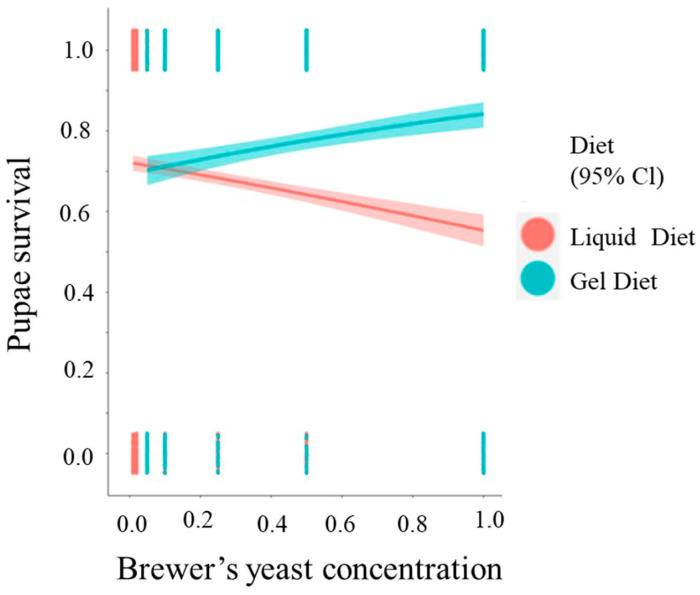
Logistic regression derived predicted probability of pupal survival in the two types of diets with 95% confidence intervals (CI), in relation to the concentration of brewer’s yeast.

Brewers’ yeast concentration (OR (95% CI) = 0.48 (0.39, 0.59), *p* < 0.001) was a significant predictor of pupal survival, unlike the diet type (OR (95% CI) = 0.87 (0.71, 1.08), *p* = 0.2) (Figure 4). The odds of pupae survival decreased by 0.7% ($e^{\log\left(0.48\right)\;0.01}$ = 0.9926872) for each additional unit percent in brewers’ yeast concentration (OR (95% CI) = 0.48 (0.39, 0.59), *p* < 0.001). The interaction between the two predictors was significant (OR (95% CI) = 4.92 (3.29, 7.41), *p* < 0.001). For the gel diets, the predicted probability of pupating increased with an increase in brewers’ yeast concentration. However, for the liquid diets the predicted probability decreased as the concentration of brewer’s yeast increased. The odds to pupate for each additional unit percent of brewer’s yeast for gel diets increase by a factor of 1.6% ($e^{log\left(4.92\right)\;0.01}=1.016)$ compared to the liquid diet. The parameter estimates (ORs) of the model are displayed in Table 3.

### 3.5. Five-Day Adults’ Survival

**Figure 5:**
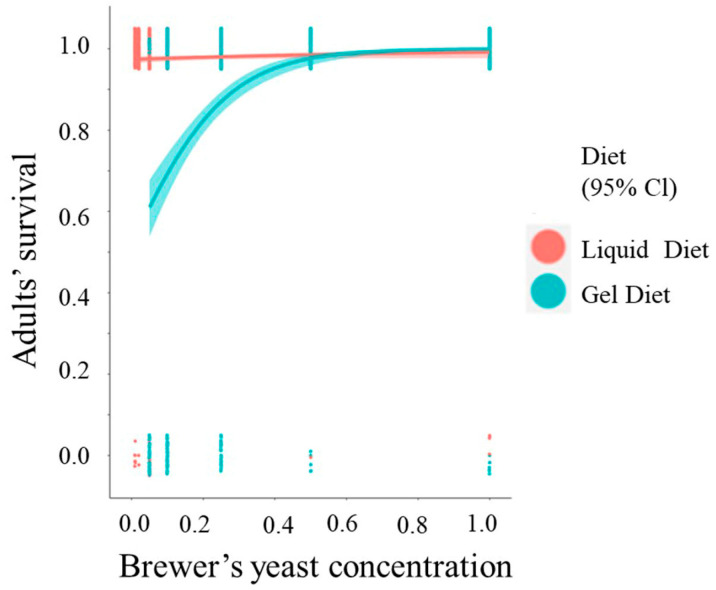
Logistic regression curves for predicted probability of adult survival in the two types of diets with 95% confidence intervals (CI) in relation to the concentration of brewer’s yeast.

The survival of adults was affected significantly by the type of diet (OR (95% CI) = 0.0683 (0.0464, 0.0993), *p <* 0.001). Specifically, the odds for adult survival when reared from the gel diet were 93.17% of those reared from the liquid diet. The survival of adults was also affected by the concentration of brewer’s yeast in each of the diets. The odds of five-day adult survival increased by 5% ($e^{log\left(143\right)\;0.01}=1.050881)$ for each additional unit percent of brewers’ yeast concentration (OR (95% CI) = 143 (51.098, 456.3858), *p <* 0.001). Table 4 presents the ORs of the model parameters. Reducing brewer’s yeast concentration by 50% on both larval diets had no effect on adult survival. Lower than 50% reduction in the content of brewer’s yeast resulted in adults that experienced high early mortality rates (Figure 5).

## 4. Discussion

Our results reveal that both liquid and gel diets could be used to successfully rear medfly from egg to adult. Reducing brewer’s yeast to 50% of its initial amount on both diet types had no negative effect on the survival and fitness of the adults. Further decreases in brewer’s yeast to levels below 50% in the gel diet resulted in growth retardation. In the gel diet, no immatures survived at the lowest concentrations of 1 and 2% of the initial amount of yeast. However, in the liquid diet, immatures survived in the lowest concentrations, and no negative effects of yeast reduction on developmental duration were found. It seems there is a strong association between brewer’s yeast availability and the diet type. This result may be based on the fact that the use of agar in the gel diet distributed the amount of brewer’s yeast evenly throughout the diet [56], in contrast with the heterogeneity of liquid diets [38]. Overall, brewer’s yeast concentration in both diets determined the immatures and adults’ fitness.

Previous studies suggested that both liquid [37,41,57,58] and gel diets [27,43] can be successfully applied for medfly mass-rearing. Liquid diets were developed to replace solid ones as they stabilize the quality of insects [42]. On the other hand, gel diets are promising for rearing of tephritids in laboratories and mass-rearing facilities [27,34,42,43,59].

Egg hatching rate was higher on the gel diet than the liquid one. Agar has the ability to modify the amount of water in the gel, providing greater water availability and higher humidity levels for eggs to hatch, while liquid diet tends to dry out faster [40]. Water, sugar and protein are evenly distributed on the gel because of agar’s physical properties [43]. However, larval developmental time was shorter on the liquid diet. Larvae sometimes may struggle to reach the nutritional components in the diet [34]. Fitness does not only depend on resource uptake but also on the bioavailability of the nutrients consumed [38]. Our liquid diet used cotton pads as bulking agent, which consisted of fibers. Aceituno-Medina et al. [38] suggested that diets containing fiber could regulate the density of nutrients independently of their proportions. Dense ingredients settle on cotton pads [34] forming clusters of protein, which could be recognized by larvae, feeding throughout development on the most nutritional spot [6]. Therefore, the clusters of protein in the liquid diets were found to promote larval development on lower concentrations. In our study, liquid diets with higher concentrations of brewer’s yeast showed lower probability of larval development. Mainali et al. [42] suggested that lower pupal numbers may reflect higher mortality on egg and larval stages due to large particle size formatted on the nutritional spots and the heterogeneity of liquid diets. Both diets seem to have positive and negative impacts on larval development, and thus, depending on the purpose of their use in the experimental process or mass-rearing procedures, they can yield the respective results.

The protein amount in larval diet regulates body size, developmental duration, survival and mating success of emerging adults [7,60]. Therefore, the reduction that we proposed on brewer’s yeast concentration could be exploited in mass-rearing facilities. Larvae feeding on higher percentages of brewer’s yeast developed faster than those feeding on lower concentrations. Kaspi et al. [61] suggested that larvae in a poor host undergo an extended development, and diets with higher protein contents are associated with faster larval development. According to Nash and Chapman [7], medfly adaptation to various nutritional environments characterizes its developmental plasticity. Increasing brewer’s yeast concentration on the gel diet led to an increased probability of pupation, whereas the same increase on the liquid diet had a negative effect. On the liquid diet, the increase in brewer’s yeast concentration resulted in lower probabilities of yielding adults. The heterogeneity that liquid diet presents on higher brewer’s yeast concentrations could cause stressful conditions and lead to lower levels of pupal survival [34]. A yeast reduction in the gel diets was also investigated by Pascacio-Villafan [27], concluding that reduced yeast concentration on the gel diets could represent a cost-effective way for mass-rearing. Brewer’s yeast reduction to 50% of our diets’ initial brewer’s yeast amount did not affect the quality of the produced insects on both types of diets in our study.

## 5. Conclusions

Both liquid and gel diets, tested in the current study, could sustain the rearing of *C. capitata*. An important requirement for the SIT is to produce qualitative insects at a lower cost. Brewer’s yeast is the most expensive ingredient on most applied diets. Lowering brewer’s yeast concentration up to 50% of the initial brewer’s yeast quantity did not affect the survival of immatures and adults on both types of diets. An increase in brewer’s yeast concentration was correlated with increased pupation and adult emergence rates in the gel type of diets. However, concerning liquid diets, some difficulties such as lower moisture retention should be overcome to be applied in a larger rearing scale. Brewer’s yeast availability is higher on liquid diets than on gel diets because of agar’s properties. Those properties make agar-based diets suitable to be applied in food-stress experimental procedures.

## Figures and Tables

**Table 1:** Composition of the liquid and the gel larval diet depending on the content of brewers’ yeast in relation to the original composition (50 g brewer’s yeast).

Diet Composition (g)							
Ingredients	Content of Brewers’ Yeast (% of the Initial Quantity)						
	100	50	25	10	5	2	1
A. Liquid diet							
Water (mL)	250	250	250	250	250	250	250
Brewer’s Yeast (g)	50	25	12.5	5	2.5	1	0.5
Soy flour (g)	25	25	25	25	25	25	25
Sugar (g)	50	50	50	50	50	50	50
Salt mixture (g)	1	1	1	1	1	1	1
Citric Acid (g)	4	4	4	4	4	4	4
Sodium propionate (g)	0.75	0.75	0.75	0.75	0.75	0.75	0.75
Ascorbic acid (g)	4	4	4	4	4	4	4
B. Gel diet B. Gel diet							
Water (ml)	250	250	250	250	250	250	250
Brewer’s Yeast (g)	50	25	12.5	5	2.5	1	0.5
Soy flour (g)	25	25	25	25	25	25	25
Sugar (g)	50	50	50	50	50	50	50
Salt mixture (g)	1	1	1	1	1	1	1
Citric Acid (g)	4	4	4	4	4	4	4
Sodium propionate (g)	0.75	0.75	0.75	0.75	0.75	0.75	0.75
Ascorbic acid (g)	4	4	4	4	4	4	4
Agar (g)	2	2	2	2	2	2	2
Nipagin (g)	0.2	0.2	0.2	0.2	0.2	0.2	0.2

**Table 2:** The estimates for the ORs of the model on effect of diet type and brewers’ yeast concentration on pupation rate.

	OR (95% CI)	*p*
(Intercept)	5.01 (4.488, 5.6166)	<0.001
Diet type (Ref: Liquid Diet)	0.0571 (0.0491, 0.0664)	<0.001
Brewer’s yeast content	0.499 (0.3966, 0.6296)	<0.001
Diet type by Brewer’s yeast content	95.7 (65.1959, 141.375)	<0.001

**Table 3:** The estimates for the ORs of the model on effect of diet type and brewers’ yeast concentration on pupal survival.

	OR (95% CI)	*p*
(Intercept)	2.59 (2.36, 2.85)	<0.001
Diet type (Ref: Liquid Diet)	0.87 (0.71, 1.08)	0.203
Brewer’s yeast content	0.48 (0.39, 0.59)	<0.001
Diet type by Brewer’s yeast content	4.92 (3.29, 7.41)	<0.001

**Table 4:** The estimates for the ORs of the model on effect of diet type and brewers’ yeast concentration on adult survival.

	OR (95% CI)	*p*
(Intercept)	25.1 (18.9797, 34.0469)	<0.001
Diet type (Ref: Liquid Diet)	0.0683 (0.0464, 0.0993)	<0.001
Brewer’s yeast content	143 (51.098, 456.3858)	<0.001

## Data Availability

Data will be made available on request.

## Supplementary Materials

The following supporting information can be downloaded at: https://www.mdpi.com/article/10.3390/insects14100828/s1, Figure S1: Age-specific cumulative curves depicting progress of pupation with 95% Confidence Intervals (CI) (A), and boxplots depicting medfly larval developmental time on the liquid and the gel diet (B).
