# Supplementary material for: Optimization of Brewer’s Yeast Quantity in Liquid and Gel Larval Diets for the Mediterranean Fruit Fly
Source: Insects. 2023 Oct 21;14(10):828. doi: 10.3390/insects14100828 (PMC10607779; doi:10.3390/insects14100828)
Supplement: Supplementary file 1 [file insects-14-00828-s001.zip › insects-2645204-supplementary.pdf]

Supplemetary Materials

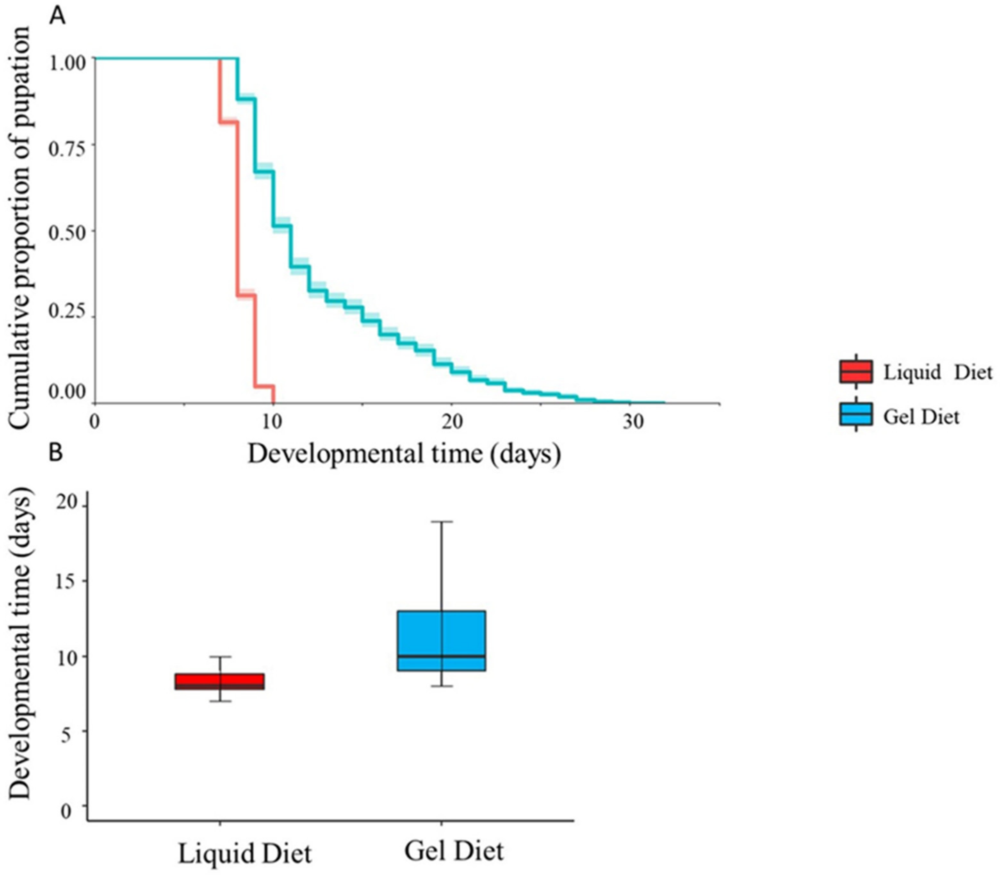

Figure S1. Age-specific cumulative curves depicting progress of pupation with 95% Confidence Intervals (CI) (A), and boxplots depicting medfly larval developmental time on the liquid and the gel diet (B).
